# Prevention of hatching of porcine morulae and blastocysts by liquid storage at 20 °C

**DOI:** 10.1038/s41598-019-42712-x

**Published:** 2019-04-17

**Authors:** Cristina A. Martinez, Josep M. Cambra, Alicia Nohalez, Inmaculada Parrilla, Jordi Roca, Jose L. Vazquez, Heriberto Rodriguez-Martinez, Maria A. Gil, Emilio A. Martinez, Cristina Cuello

**Affiliations:** 1 Department of Medicine and Animal Surgery, Faculty of Veterinary Medicine, International Excellence Campus for Higher Education and Research “Campus Mare Nostrum”, University of Murcia, 30100 Murcia, Spain; 2 Institute for Biomedical Research of Murcia (IMIB-Arrixaca), Campus de Ciencias de la Salud, Carretera Buenavista s/n, 30120 El Palmar, Murcia Spain; 3 Department of Clinical & Experimental Medicine (IKE), Linköping University, Campus US, 58183 Linköping, Sweden

**Keywords:** Animal biotechnology, Medical research

## Abstract

Vitrification is the ideal method for long-lasting storage of porcine embryos. However, both strict airline regulations for transport of liquid nitrogen dewars and the technical problems experienced when vitrified embryos are transferred using non-surgical procedures have led to the introduction of alternative storage methods, such as preserving embryos in liquid state. This study evaluated whether a pH-stable medium containing high concentrations of either foetal calf serum (FCS; 50%) or BSA (4%) combined with storage at temperatures of 17 °C or 20 °C maintained *in vivo*-derived morulae and blastocysts alive and unhatched (a sanitary requirement for embryo transportation) during 72 h of storage. Neither FCS nor BSA supplements were able to counteract the negative effect of low temperatures (17 °C) on embryonic survival after storage. At 20 °C, the protective effect of FCS or BSA depended on embryo stage. While FCS successfully arrested embryo development of only blastocysts, BSA arrested the development of both morulae and blastocysts. Over 80% of BSA arrested embryos restarted development by conventional culture and progressed to further embryonic stages, including hatching. In conclusion, porcine morulae and blastocysts can survive and remain unhatched during at least 72 h when stored at 20 °C in a BSA-containing medium.

## Introduction

Implementing embryo transfer (ET) technology in commercial pig breeding should have major productive and economic gains. Genetic dissemination through ET of morulae or blastocysts with intact zona pellucida (ZP) is unquestionably of major benefit for the sector because it markedly reduces sanitary risks and avoids current animal welfare issues associated with the costly shipping of live animals^1^.

Porcine ET has advanced considerably over the last years, overcoming the most traditional problems associated with the technology, particularly those related to the development of nonsurgical deep uterine (NsDU) ET techniques and improvements in embryo preservation^1^.

Although cryopreservation is the ideal procedure for long-term embryo storage, allowing safe worldwide transportation, its use has lately been jeopardized by the strict rules applied for the commercial air transport of liquid nitrogen (LN_2_) dewars. Moreover, and perhaps more importantly, NsDU-ET requires far more cryopreserved embryos than surgical ET to achieve similar fertility, thus decreasing its effectivity^2^. These disadvantages have led to a demand for alternative storing methods, such as embryo preservation in liquid state. A primary condition for liquid storage is that the embryos are transferred at morula or unhatched blastocyst stages because the NsDU-ET is performed in the middle or the anterior quarter of a uterine horn. We should remember that under conventional culture conditions [i.e., media containing bovine serum albumin (BSA) or foetal calf serum (FCS) at 38.5 °C and 5% CO_2_ in air], a high percentage of *in vivo*-derived morulae (20%) and blastocysts (50%) hatches after 24 h of culture^3,4^. This fact excludes these conventional culture conditions from being used as a procedure for embryo storage because they favour hatching, thus jeopardizing the sanitary covering of the embryos for transport and ET^5^.

In a previous study, we stored porcine morulae at 37 °C for 24 h in a pH-stable medium without CO_2_ gassing. More than 95% of these embryos progressed to the unhatched blastocyst stage, and although there was a certain delay in embryo development during storage, the resulting blastocysts retained a similar potential to develop to term compared to unstored blastocysts^4^. This storage period should be long enough to enable transport of the embryos in liquid state from the donor to the recipient farms, at least at regional and national levels, considering most medium-size countries. However, a longer storage period would allow for transport over longer hauls including trans-oceanic movement, and would therefore represent a more rational use of the ET technology. In another recent study, we evaluated the potential of prolonging the liquid-storage period for up to 72 h after collection. Morulae stored at 37 °C^6^ and blastocysts stored at 25 °C (unpublished data) in a semi-defined medium containing 0.4% BSA maintained their *in vitro* viability and developmental competence for up to 72 h. However, although these embryos exhibited an important development delay compared to controls, many of them hatched after 72 h of storage. Additional efforts are thus needed to identify alternative storing conditions that would allow for overseas shipment of un-hatched embryos in a liquid state.

Cooling to refrigeration temperatures (around 4–5 °C) not only inhibits embryo metabolism but also maintains embryo viability in several species, including bovine^7–9^, ovine^10^, rabbit^11,12^, and rodent^13–15^ species and humans^16^. However, such cooled storage shows that embryonic survival rates differ among species, embryo stages and laboratory procedures. Noteworthy, few studies of the effect of hypothermic temperatures on the survival of porcine preimplantation embryos have been conducted. It is widely known that porcine embryos contain high amounts of cytoplasmic lipids^17,18^, which provides them with substantial sensitivity to temperatures below 15 °C^19^. Moreover, a storage temperature of 18 °C not only delays embryo development but also results in much higher embryo degeneration rates than those observed at 25 °C or 38 °C^20^.

The media used for *in vitro* embryo culture are frequently supplemented with serum or serum-derivate (e.g., BSA) as protein source to facilitate embryo manipulation. In addition, these supplements are also included in cryopreservation media. BSA protects the cell membranes of both embryos^21^ and spermatozoa^22–24^ during cryopreservation, and increases survival and development rates of porcine embryos when stored in a liquid state^6^. In those studies, survival and hatching rates of embryos stored at 37 °C (morulae) or 25 °C (blastocysts) in medium supplemented with PVA were substantially lower than those obtained using the same medium supplemented with 0.4% BSA. On the other hand, the benefits of serum supplementation for embryo cryopreservation seem to depend on both species and storage temperature. While it does exert different effects on bovine embryo cryotolerance^25–28^, serum appears to confer increased cryotolerance to porcine embryos^29^. The effect of serum on bovine embryos stored at refrigeration temperatures is clearer; supplementing saline medium with 10% FCS prolonged their survival while maintained at 4 °C from 1 to 3 days^30^. Moreover, a saline medium supplemented with 50% FCS allowed bovine embryos to be stored for up to 7 days at 4 °C without affecting pregnancy rates after ET or the later health of the obtained offspring^9^.

The objectives of the present experiments were designed to test the hypothesis that porcine embryos can survive at hypothermic temperatures when maintained in saline media containing high concentrations of FCS or BSA. Both *in vitro* viability and developmental capacity of *in vivo*-derived pig embryos at morula or unhatched blastocyst stages were evaluated after storage for 72 h in CO_2_-free media supplemented with 50% FCS or 4% BSA, at either 17 °C or 20 °C.

## Results

### Experiment 1

This experiment examined the effects of storage temperature and medium supplementation on the viability and embryonic development of compacted morulae stored for 72 h in a total of three replicates. Immediately after collection, groups of 7 to 10 morulae were stored for 72 h at 17 °C or 20 °C in pH-stable NCSU-23 medium containing 10 mM HEPES and supplemented with 50% FCS or 4% BSA in a 2 × 2 factorial design. At the end of storage, both embryonic viability and morphological development were assessed. Then, the stored embryos were conventionally cultured for an additional period of 48 h to reassess viability and development and to evaluate their hatching competence. Fresh (non-stored) morulae were cultured under conventional conditions for 48 h and used as control groups. Embryos were obtained from 12 donors and embryos from each donor were equally and randomly allocated to each of the groups. The mean number of corpora lutea was 19.7 ± 4.1 (range 12 to 25 corpora lutea). The recovery and fertilization rates were 90.2% and 92.9%, respectively. A total of 177 morulae were used in this experiment.

The rates of embryonic viability after 72 h of storage under different temperatures and in different media conditions are shown in Table 1. Neither the FCS nor the BSA were able to counteract the negative effect of the lowest temperature (17 °C) on embryonic survival during storage. Only 52.4% to 59.1% of morulae stored in the presence of 50%-FCS or 4%-BSA were viable after 72 h of preservation, and this percentage decreased and reached 40% to 45% after 48 h in the following conventional culture. These percentages were much lower (P < 0.001) than those observed in the controls (97.6%).

**Table 1:** Survival rates of *in vivo*-produced porcine morulae after 72 h of storage at different temperatures and in different medium supplementations.

Group	Storage temperature	Number of embryos	72 h of storage [Number of embryos (%)]		48 h of conventional culture [Number of embryos (%)]^#^		
			Viable	Degenerated	Viable	Degenerated	Cell numbers per blastocyst
50%-FCS	17 °C	21	11 (52.4)^a^	10 (47.6)^a^	8 (38.1)^a^	13 (61.9)^a^	63.8 ± 6.8^a^
4%-BSA		22	13 (59.1)^a^	9 (40.9)^a^	10 (45.4)^a^	12 (55.6)^a^	64.5 ± 13.1^a^
50%-FCS	20 °C	48	48 (100)^b^	0 (0.0)^b^	30 (62.5)^a^	18 (37.5)^a^	61.4 ± 9.9^a^
4%-BSA		45	44 (97.8)^b^	1 (2.2)^b^	37 (82.2)^b^	8 (17.8)^b^	70.1 ± 9.2^a^
Control^##^		41	—	—	40 (97.6)^c^	1 (2.4)^c^	114.4 ± 19.8^b^

^#^After storage, the viable stored embryos from each group were`ultured for an additional 48 h under conventional culture conditions (NCSU-23 culture medium supplemented with 0.4% BSA and 10% FCS, at 38.5 °C in humidified air with 5% CO_2_) to reassess their *in vitro* survival. ^##^Control morulae were cultured under conventional conditions for 48 h. ^a,b,c^Different superscripts in the same column represent significant differences (P < 0.05).

In contrast, there were no apparent signs of degeneration at the end of storage in most embryos stored at 20 °C. Following incubation under conventional culture conditions, more viable embryos were present in the 4%-BSA group than in the 50%-FCS group (82.2% vs. 62.5%, respectively; P < 0.05), but survival rates were lower (P < 0.05) in either group compared to controls.

**Figure 1:**
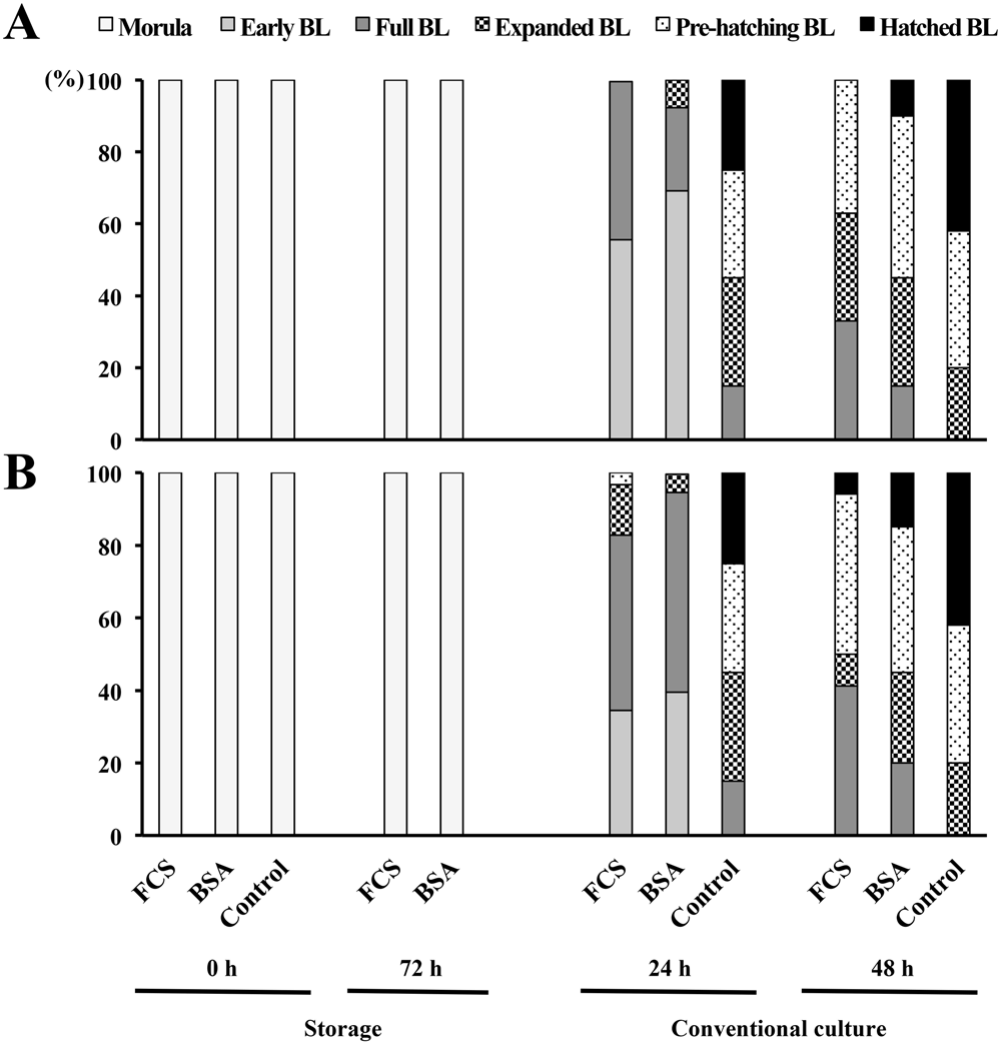
Embryonic progression of *in vivo*-produced porcine morulae stored for 72 h at different temperatures and medium supplementations. (**A**) Bar graphs showing the proportion of embryos that reached each developmental stage per group after 72 h of storage at 17 °C and after 24 h and 48 h of conventional culture (NCSU-23 culture medium supplemented with 0.4% BSA and 10% FCS at 38.5 °C in humidified air with 5% CO2). (**B**) Bar graphs showing the proportion of embryos that reached each developmental stage per group after 72 h of storage at 20 °C and after 24 h and 48 h of conventional culture. BL: blastocyst.

Embryonic development was arrested at the morula stage in both experimental groups after 72 h of storage regardless of medium supplementation or storage temperature. However, surviving embryos were able to restart development under conventional culture conditions; most of them achieving early-full blastocyst or expanded-pre-hatching blastocyst stages after 24 h and 48 h, respectively (Table 2). Nevertheless, development was slower in stored embryos than in control embryos (P < 0.001), where most embryos were from the expanded to hatched or from the pre-hatching to hatched blastocyst stages after 24 h and 48 h under conventional culture conditions, respectively (Fig. 1). There were no differences between 50%-FCS and 4%-BSA groups regarding total cell numbers per blastocyst among the surviving stored embryos, following 48 h of conventional culture, but the number of cells per embryo in the experimental groups (66.8 ± 12.4) was almost half than in the control embryos (114.4 ± 19.8) (P < 0.001).

**Table 2:** Embryonic developmental stages of *in vivo*-produced porcine morulae after 72 h of storage at different temperatures and in different medium supplementations.

Group	Storage temperature	Embryo stage^#^ [N (mean ± SD)]			
		Storage		Conventional culture^##^	
		0 h	72 h	24 h	48 h
50%-FCS	17 °C	21 (1.0 ± 0.0)	11 (1.0 ± 0.0)	9 (2.4 ± 0.5)^a^	8 (4.0 ± 1.1)^a^
4%-BSA		22 (1.0 ± 0.0)	13 (1.0 ± 0.0)	13 (2.4 ± 0.6)^a^	10 (4.5 ± 1.1)^a^
50%-FCS	20 °C	48 (1.0 ± 0.0)	48 (1.0 ± 0.0)	34 (2.9 ± 0.8)^a^	30 (4.1 ± 1.0)^a^
4%-BSA		45 (1.0 ± 0.0)	44 (1.1 ± 0.3)	40 (2.6 ± 0.6)^a^	37 (4.5 ± 1.0)^a^
Control^###^		41 (1.0 ± 0.0)	—	40 (4.7 ± 0.7)^b^	40 (5.3 ± 0.5)^b^

^#^The developmental stage was scored from 1 to 6 as: 1, morula; 2, early blastocyst; 3, full blastocyst; 4, expanded blastocyst; 5, pre-hatching blastocyst; and 6, hatching and hatched blastocyst. ^##^After storage, the embryos from each group were cultured under conventional conditions (NCSU-23 medium supplemented with 0.4% BSA and 10% FCS at 38.5 °C in humidified air with 5% CO_2_) for up to 48 h to re-evaluate the embryonic development. ^###^Controls included morulae cultured under conventional conditions for up to 48 h. ^a,b^Different letters in the same column indicate significant differences (P < 0.01). Values are expressed as mean ± SD.

### Experiment 2

This experiment evaluated the effects of storage temperature and medium supplementation on the viability and embryonic development of unhatched blastocysts stored for 72 h (three replicates) following the same experimental design as described for Experiment 1 except that stored blastocysts were conventionally cultured for an additional period of only 24 h. and controls embryos were fresh (non-stored) unhatched blastocysts cultured under conventional conditions for 24 h.

Embryos at the blastocyst stage were collected from 13 sows. The mean number of corpora lutea in the donor sows was 23.7 ± 5.2 (range 15 to 32 corpora lutea). Embryo recovery rate was 94.5%, and fertilization rate reached 92.8%.

**Figure 2:**
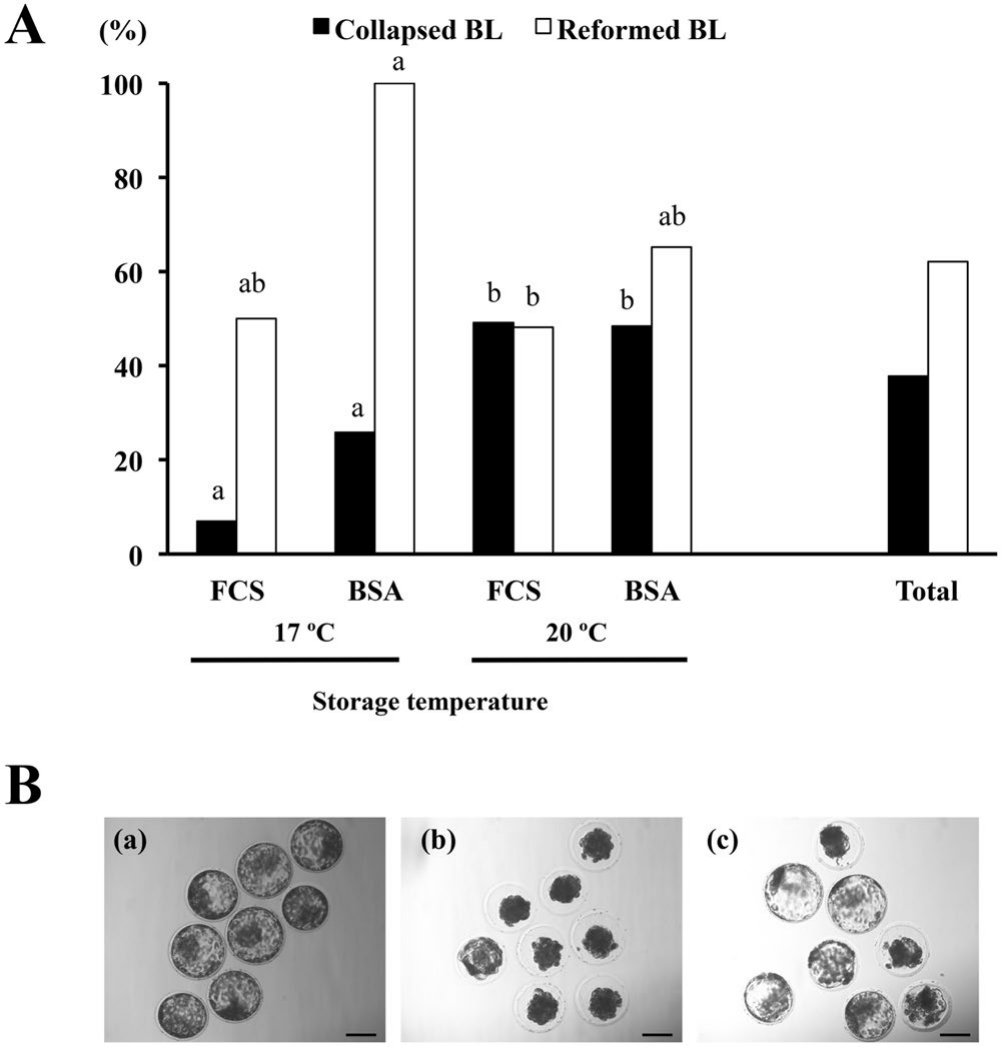
Collapse and re-expansion of blastocysts. (**A**) The proportion of blastocysts that spontaneously collapsed after 72 h of storage at different temperatures and in different medium supplementations and the re-expansion ability of the collapsed blastocysts after 24 h of conventional culture (NCSU-23 culture medium supplemented with 0.4% BSA and 10% FCS at 38.5 °C in humidified air with 5% CO_2_). ^a,b^Different letters shown per variable (collapsed or reformed) indicate significant differences (P < 0.05). BL: blastocyst. (**B**) Representative pictures of blastocyst collapse and re-expansion. (a) blastocysts just after collection; (b) selected blastocysts collapsed after 72 h of storage; (c) re-expansion of blastocysts after 24 upon conventional culture. Scale bar is 100 µm.

Data related to the *in vitro* survival of the blastocysts stored for 72 h are shown in Table 3. By the end of storage, some blastocysts spontaneously collapsed (Fig. 2). The percentage of these blastocysts was below 26% at 17 °C while close to 50% at 20 °C in the experimental groups, regardless of the supplementation used in the media (P < 0.05). Collapsed blastocysts were not observed in the control group. More than 60% of the collapsed blastocysts had reformed their blastocoel and showed normal morphology after 24 h of conventional culture. These reformed blastocysts were considered viable embryos. The probability of reforming the blastocoel was higher (P < 0.05) in blastocysts stored in medium containing 4%-BSA than in those in medium containing 50%-FCS (73.0% vs. 48.2%, respectively).

**Table 3:** Survival rates of *in vivo*-produced porcine blastocysts after 72 h of storage at different temperatures and in different medium supplementations.

Group	Storage temperature	Number of embryos	End of storage [Number of embryos (%)]			24 h of conventional culture [Number of embryos (%)]^#^			
			Viable	Collapsed	Degenerated	Viable	Collapsed	Degenerated	Cell numbers per blastocyst
50%-FCS	17 °C	29	6 (20.7)^a^	2 (6.9)^a^	21 (72.4)^a^	7 (24.1)^a^	1 (3.4)	21 (72.4)^a^	86.4 ± 9.0^a^
4%-BSA		31	5 (16.1)^a^	8 (25.8)^a^	18 (58.1)^a^	13 (41.9)^a^	0 (0.0)	18 (58.1)^a^	90.3 ± 12.2^a^
50%-FCS	20 °C	55	27 (49.1)^b^	27 (49.1)^b^	1 (1.8)^b^	40 (72.7)^b^	1 (1.8)	14 (25.4)^b^	90.1 ± 17.3^a^
4%-BSA		60	27 (45.0)^b^	29 (48.3)^b^	4 (6.7)^b^	48 (80.0)^b^	0 (0.0)	112 (20.0)^b^	92.9 ± 13.3^a^
Control^##^		49	—	—	—	48 (97.9)^c^	0 (0.0)	1 (2.1)^c^	131.2 ± 11.9^b^

^#^After storage, the viable stored embryos from each group were cultured for an additional 24 h under conventional culture conditions (NCSU-23 culture medium supplemented with 0.4% BSA and 10% FCS, at 38.5 °C in humidified air with 5% CO_2_) to reassess their *in vitro* survival. ^##^Control blastocysts were cultured under conventional conditions for 24 h. ^a,b,c^Different superscripts in the same column represent significant differences (P < 0.05).

By the end of storage, above 55% of the blastocysts maintained at 17 °C showed clear signs of degeneration, with no differences between groups. In contrast, the degeneration rate observed in blastocysts stored at 20 °C was lower than 7% in both groups. After 24 h in conventional culture, survival rates of blastocysts were significantly lower for those stored at 17 °C (24.1% to 41.9%) than those stored at 20 °C (72.7% to 80.0%) (P < 0.005), regardless of the supplementation used in the media. Control blastocysts had the highest survival rate (97.9%) by 24 h of conventional culture (P < 0.001).

**Figure 3:**
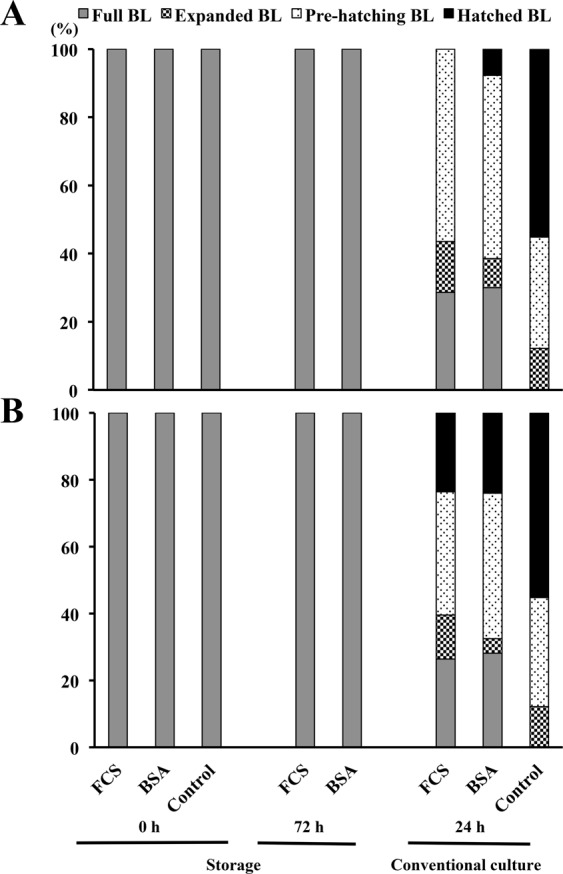
Progression stages of *in vivo*-produced porcine blastocysts stored for 72 h at different temperatures and in different medium supplementations. (**A**) Bar graphs showing the proportion of embryos that reached different developmental stages per group after 72 h of storage at 17 °C and after 24 h and 48 h of conventional culture (NCSU-23 culture medium supplemented with 0.4% BSA and 10% FCS at 38.5 °C in humidified air with 5% CO2). (**B**) Bar graphs showing the proportion of embryos that reached different developmental stages per group after 72 h of storage at 20 °C and after 24 h and 48 h of conventional culture. BL: blastocyst.

There were no morphological changes in blastocysts between the beginning and the end of the period of storage, regardless of the temperature or supplementation used (Table 4). As seen among embryos at the morula stage, surviving blastocysts also restarted their development when placed in conventional culture. After 24 h of conventional culture, embryonic development did not differ between experimental groups, regardless of the temperature used (Table 4). However, the surviving embryos in the experimental groups presented a clear developmental delay (P < 0.001) compared to the surviving control embryos, where most of embryos were at the pre-hatching or the hatched blastocyst stage at 24 h of conventional culture (Fig. 3). Additionally, a significant reduction was seen in total cell numbers per blastocyst between stored blastocysts and controls, after 24 h in conventional culture (89.2 ± 14.9 and 131.2 ± 11.9 cells, respectively).

**Table 4:** Embryonic developmental stages of *in vivo*-produced porcine blastocysts after 72 h of storage at different temperatures and in different medium supplementations.

Group	Storage temperature	Embryo stage# [N (mean ± SD)]		
		Storage		Conventional culture^##^
		0 h	72 h	24 h
50%-FCS	17 °C	29 (3.0 ± 0.4)	6 (3.1 ± 0.2)	7 (4.3 ± 1.0)^a^
4%- BSA		31 (3.0 ± 0.0)	5 (3.0 ± 0.0)	13 (4.4 ± 1.3)^a^
50%-FCS	20 °C	55 (2.9 ± 0.4)	27 (3.0 ± 0.7)	38 (4.6 ± 1.2)^a^
4%-BSA		60 (2.9 ± 0.4)	27 (3.1 ± 0.7)	46 (4.6 ± 1.2)^a^
Control^###^		49 (3.0 ± 0.3)	—	48 (5.4 ± 0.1)^b^

^#^The developmental stage was scored from 1 to 6 as: 2, early blastocyst; 3, full blastocyst; 4, expanded blastocyst; 5, pre-hatching blastocyst; and 6, hatching and hatched blastocyst. ^##^After storage, the embryos from each group were cultured under conventional conditions (NCSU-23 medium supplemented with 0.4% BSA and 10% FCS at 38.5 °C in humidified air with 5% CO_2_) for up to 48 h to re-evaluate their embryonic development. ^###^Controls included blastocysts cultured under conventional conditions for up to 48 h. ^a,b^Different letters in the same column indicate significant differences (P < 0.01). Values are expressed as mean ± SD.

**Figure 4:**
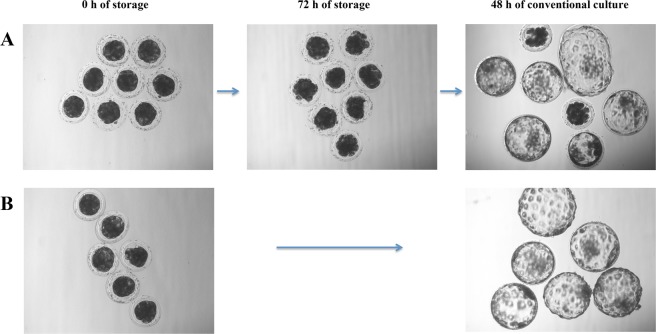
Preservation of porcine *in vivo*-derived morulae. (**A**) Representative images showing morulae at 0 h and 72 h of storage in medium containing 4% BSA at 20 °C or after 48 h upon conventional culture (NCSU-23 culture medium supplemented with 0.4% BSA and 10% FCS at 38.5 °C in humidified air with 5% CO2). (**B**) Representative images showing morulae at 0 h and 48 h of conventional culture (controls). Scale bar is 100 µm.

Representative pictures of the *in vitro* developmental capacity of the embryos preserved in medium supplemented with 4%-BSA at 20 °C for 72 h are shown in Fig. 4.

## Discussion

This is the first study to show that *in vitro* development of *in vivo*-derived porcine morulae and unhatched blastocysts can be successfully arrested for at least 72 h if stored at 20 °C in a 4%-BSA-containing medium. More than 80% of the arrested embryos were able to restart development and progress to a further embryonic stage when cultured under conventional conditions.

In this study, in addition to the reasons indicated in previous reports^4,6^, we used embryos at the morula and blastocyst stages because these are the most appropriate stages to practically perform the current NsDU-ET procedure^31,32^. From a practical point of view, and to facilitate potential future transportation conditions, the embryos were hereby stored in a pH-stable medium without CO_2_ gassing, as this approach was successfully used in earlier studies for liquid storage of porcine morulae^4,6^. Additionally, the storage period was set at 72 h because this period should be sufficient to ensure any worldwide distribution of the embryos. We decided to use storage temperatures of 17 °C and 20 °C in an attempt to decrease the metabolism of the embryos while preserving their viability and thereby ensuring they would remain unhatched until the end of the storage period. We supplemented the storage medium with FCS or BSA at concentration much higher than routine, because of their beneficial effects on cold tolerance in bovine embryos^9,24,30^. This protective role of either of these components could be of particular importance for porcine embryos because they are extremely sensitive to temperatures below 18 °C^19,20^.

When embryos were stored at 17 °C, survival rates at both the end of the conservation period and after the conventional culture were dramatically reduced. Embryo degeneration occurred independent of the stage of development and the type of supplement used. These data clearly demonstrate that neither 50%-FCS nor 4%-BSA supplementation were effective in protecting morulae and blastocysts from moderate cooling temperatures and confirm the extreme sensitivity of porcine embryos to temperatures below 18 °C, as previously reported^19,20^.

In contrast, survival rates were high by the end of storage when morulae were stored at 20 °C. However, unlike the control embryos, a variable percentage of morulae classified as viable after storage showed clear signs of degeneration following conventional culture; this was not surprising because we also obtained high survival rates in morulae stored for 48 h at 25 °C, and more than 20% of these embryos did degenerate during conventional culture in a previous study^6^. Determining survival and degeneration rates was more complicated for embryos in the blastocyst stage because almost 50% of the blastocysts stored at 20 °C appeared collapsed by the end of storage and could only be classified as viable or degenerate, depending on whether they were able (or not) to reform the blastocoel by the end of the conventional culture period. Anyway, our results suggest that some embryos with apparently normal morphology post-storage were functionally damaged during the process, which agrees with previous studies^6^ regarding the importance to use a period of conventional culture after storage to evaluate the functionality of any stored embryos.

At 20 °C, the protective effect of 50%-FCS or 4%-BSA depended on embryo stage. While embryo survival rate after 48 h of conventional culture was higher for morulae in the 4%-BSA group than in those in the 50%-FCS group, there were no significant differences in survival rates between blastocysts from any group. These results suggest that blastocysts are less sensitive than morulae to a temperature of 20 °C. The following findings support this hypothesis: (i) porcine embryo cryotolerance depends on the developmental stage of the embryos, with those in more advanced stages (e.g., hatched and expanded blastocysts) showing greater cryotolerance than those in less advanced stages^33,34^) and (ii) in another study, while more than 95% of blastocysts were successfully preserved in liquid state for 48 h at 25 °C (unpublished data), only 75% of the morulae survived these conditions^6^.

Our results also suggest that 4%-BSA exerts a stronger protective effect than 50%-FCS on embryos stored at 20 °C, at least at the morula stage. These results are contrary to those reported in bovine, where embryo survival rate after storage at 4 °C for 72 h was higher for embryos stored in medium containing 50% FBS than in those stored in either low or high concentrations of BSA^9^. Moreover, those authors obtained high *in vitro* and *in vivo* survival rates in blastocysts stored for 7 days at 4 °C in a medium with a high serum concentration (50%), and they found that the presence of serum during chilling preservation, unlike during embryo culture^35,36^, did not alter either embryonic or foetal development. This apparent discrepancy might be simply due to differences between species. In support of this hypothesis, it has been reported that in bovine, low concentrations of serum may be a better choice than BSA for embryo culture systems, while in pigs, addition of BSA seems to be superior to serum^37^.

Although several reports have demonstrated the numerous benefits of BSA for cell and embryo culture^37–39^, little information is available about the direct effects of BSA on embryo preservation. BSA has been shown to confer tolerance to strong hypothermia (4 °C) of ovine embryos^40^ and to increase the ability of bovine blastocysts to survive cryopreservation^21^. Recently, the beneficial effects of BSA on the liquid preservation of porcine embryos was clearly demonstrated by our laboratory^6^. In agreement, the results of the present study further suggest that 4%-BSA exerts a protective effect when the temperature of storage is 20 °C; an approach that allowed a high percentage (>80%) of morulae and blastocysts to survive storage for 72 h and further to maintain functionality upon conventional culture conditions. Although it has been hypothesized that the non-permeable large molecule BSA may interact with the cell membrane and modulate its structure and function^40^, the cryo-protective mechanisms of BSA on embryos at hypothermic temperatures remain to be determined.

Studies performed in rodents, ruminants and equine have indicated that low, supra-zero temperatures (around 4 °C) induce a temporary arrest of embryonic development^9,12,13,15,41^. Interestingly, our results show that porcine embryos (morulae and blastocysts) did not grow during the 72 h of storage tested, at either 17 °C or 20 °C. This is not surprising since in our previous work, we observed a certain development delay in porcine morulae preserved for up to 72 h at 25 °C or 37 °C, with the delay being most marked at 25 °C^4,6^. Our data suggest that the metabolism of pig embryos is severely reduced at temperatures below 20 °C, an effect that could be related to the misregulation of genes that play important roles in embryo development and cell proliferation and alterations in other molecular events, as previously reported for mice embryos stored at refrigeration temperatures^13,15^. Although the developmental arrest observed in the stored embryos was temporal, their final developmental stage was equally delayed including a lower total cell number compared to controls. However, this situation may not be relevant for two main reasons; firstly, we previously reported that developmentally delayed stored pig embryos retained the capacity to develop *in vivo*^4,6^; and secondly, although the relevance of a cell number limit is not yet fully understood, embryos with significantly lower cell numbers do not exhibit a compromised implantation capacity, at least in rodents^15^.

Studies from our laboratory have indicated that at 25 °C, a proportion of the stored embryos hatched after 72 h in storage (unpublished data). These embryos must be eliminated from eventual transport because an intact ZP is indispensable for preserving correct sanitary conditions for the embryos^5^. The fact that a temperature of 20 °C maintains the embryos in a developmentally repressed yet viable state is particularly important because it opens the door to extend the storage period for both morula- and blastocyst-stage embryos, thus avoiding the loss of embryos by hatching.

In the present study, the ability of stored embryos to hatch upon conventional culture was lower than that observed in control embryos. While 40% of the control morulae hatched after 48 h in conventional culture, less than 15% of the stored embryos hatched. A similar pattern was observed in blastocysts after 24 h of conventional culture. Because the hatching rate is a good biomarker of the potential quality and developmental capacity of blastocysts^42^, we speculate that the functionality of preserved embryos could be impaired by storage conditions. However, we have to assume that the short conventional culture performed in this study (48 h for morulae and 24 h for blastocysts) was likely not enough to compensate for the developmental delay caused by the storage. In support of this hypothesis, the hatching rates of conventionally cultured morulae increased from 30% to 80% at 48 h and 72 h of culture, respectively^6^. More studies are needed to confirm the functional and *in vivo* developmental ability of stored morulae and blastocysts under our conditions of study.

Surprisingly, while control blastocysts did not show any sign of collapse during culture, almost 50% of those blastocysts stored at 20 °C were classified as collapsed by the end of storage. After 24 h of conventional culture, however, 60% of these blastocysts showed re-expansion of the blastocoel and recovered a typical morphology, indicating they were alive. The collapse and re-expansion of a blastocyst is not an uncommon phenomenon, and it has been reported in several species, including the pig^43^. In human, blastocysts can collapse in response to several factors, including changes in temperature or incubation media or simply mechanically-induced through aspiration into a pipette^44^. Likewise, our studied stored blastocysts, unlike the controls, were handled in a different culture medium (conventional medium) at a different temperature (38.5 °C) and were aspirated with a pipette from the storage tubes to the conventional culture Petri dish. Many blastocysts collapsed during the examination, indicating any of these stressful environmental factors could be, isolated or concerted, responsible for blastocyst collapse. Whether collapse is a sign of compromised embryo functionality is still discussed. In human blastocysts, collapse has been related to lower implantation success^45^, but others report that artificial collapse improved clinical outcomes in fresh blastocyst transfer cycles^46^. Moreover, blastocyst collapse is frequently used prior to vitrification and considered to improve its effectiveness^47,48^.

In conclusion, while FCS-containing medium was able to successfully arrest embryo development for at least 72 h of only unhatched blastocysts at 20 °C, BSA in the medium arrested the development of both morulae and unhatched blastocysts. In more than 80% of the morulae and blastocysts stored at 20 °C in a medium containing a 4%-concentration of BSA, development was arrested for at least 72 h without compromising further viability upon conventional culture. Notably, this developmental arrest could extend the storage period while maintaining the embryos in the unhatched stage, a sanitary requirement for ET. Although, under these conditions, FCS in the medium was also able to arrest the development of unhatched blastocysts, the use of a BSA-containing medium is highly recommended owing to its lower impact on animal welfare issues, when considering the controversial animal source for the FCS. Although the results of the present study offer new opportunities for the preservation of porcine embryos in liquid state, further research is necessary to evaluate their *in vivo* developmental ability. Liquid state embryo preservation, when optimized, may constitute an alternative method to vitrification for medium-term porcine embryo storage because it avoids the strict regulatory rules associated with flight transportation of LN2 and, more importantly, the persisting procedural complications related to the combined use of NsDU-ET and vitrified embryos.

## Methods

All chemical reagents were acquired from Sigma-Aldrich Quimica SA (Madrid, Spain) unless otherwise stated. Animal procedures were performed according to the European Directive 2010/63/EU EEC for animal experiments and were previously examined and approved by the Ethical Committee for Experimentation with Animals, University of Murcia and the Ministry of Agriculture and Water, Region of Murcia (Spain) (research code: 183/2015).

### Animals

A total of 25 crossbred sows (Landrace × Large-White; parity 2 to 6) with similar lactation periods (21 to 24 d) were randomly selected at weaning and allotted to the experiments. Mature boars (2 to 3 years of age) housed at a breeding artificial insemination (AI) station were used as semen providers. All animals had free access to water and were fed commercial diets according to their nutrient requirements.

### Artificial insemination and embryo recovery

The sows were checked for signs of oestrus once a day, beginning on the day of weaning, as previously described^6^. The day of onset of oestrus was considered day 0 (D0) of the cycle. Only sows with 4 or 5 days of weaning-to-oestrus interval were selected for the experiments. Sows were post-cervically inseminated 6 and 24 h after onset of oestrus, as described by Martinez *et al*.^2^.

Laparotomy was performed to collect morulae (D5 of the cycle) or blastocysts (D6) following the procedure described by Angel *et al*.^31,32^. Briefly, after sedation with azaperone (2 mg/kg body weight, intramuscular), donor sows were anaesthetised with sodium thiopental (7 mg/kg body weight, intravenous) followed by isoflurane-maintained narcosis (3 to 5%). Embryos were collected by flushing the tip of each uterine horn with modified Tyrode’s lactate (TL)-HEPES-polyvinyl alcohol (PVA) medium (TL-PVA). Recovery rate (the ratio of embryos and oocytes collected to the total number of corpora lutea x100) and fertilization rate (the ratio of viable embryos to the total number of embryos and oocytes recovered x100) were assessed. Only compacted morulae and unhatched blastocysts with excellent or good morphology according to the standards of the International Embryo Transfer Society^49^ were selected for the experiments.

### Embryo storage, embryo culture and evaluation of viability, embryonic developmental stage and total cell numbers

Following washing in TL-PVA medium, the embryos were stored for 72 h at two different temperatures (17 °C or 20 °C) and in media differentially supplemented (50% FCS or 4% BSA) as per the experimental design. At the end of storage time, the embryos were assessed for viability and development stage progression using a stereomicroscope. Then, the embryos were immediately cultured to re-evaluate viability and embryonic development under conventional conditions in 500 μL of NCSU-23^50^ supplemented with 0.4% BSA and 10% FCS at 38.5**°**C in 5% CO_2_ in air with saturated humidity for an additional 24 h or 48 h. Embryos with either adequate morphology or whose development progressed to further stages during storage or while in conventional culture were considered viable. The *in vitro* survival rate was calculated as the ratio of viable embryos to the total number of embryos evaluated x 100.

The developmental stages of the embryos were scored from 1 to 6 according to the following previously described morphological criteria^4^: 1, compacted morula (compacted blastomeres and an unrecognizable cell periphery); 2, early blastocyst (incipient discernible blastocoel); 3, full blastocyst (differentiated inner cell mass and trophoblast); 4, expanded blastocyst (full blastocysts with an increased diameter and thinned ZP); 5, pre-hatching blastocyst (expanded blastocysts with an extremely thin ZP); and 6, hatching or hatched blastocyst (blastocyst with broken or lost ZP). The hatching rate was calculated as the ratio of hatching or hatched blastocysts to the total number of embryos evaluated x 100.

Total cell numbers per blastocyst were determined as previously described^51^. Briefly, the embryos were fixed in 4% paraformaldehyde for 30 min, washed twice with PBS supplemented with 3 mg/mL BSA and mounted in 4 µL of Vectashield (Vector, Burlingame, CA, USA) containing 10 µg/mL Hoechst 33342. The embryos were then photographed using a fluorescence microscope (excitation filter: 330 to 380 nm), and the total number of cells with nuclei showing blue fluorescence was counted.

### Statistics

The data were analysed with the IBM SPSS 24.0 Statistics package (IBM, Chicago, IL, USA). Percentage data were compared using Fisher’s exact test. Scored data (i.e., embryonic developmental stage) were analysed using the Kruskal–Wallis test and, if necessary, two by two comparisons for two independent samples with the Mann–Whitney U-test. The total cell number per blastocyst was analysed to evaluate normality and homogeneity of variances by the Kolmogorov–Smirnov and Levene tests, respectively, and groups were compared by a mixed-model ANOVA. The ANOVA model included the main effects of temperature and supplementation and their interactions and the random effect of the replicate. When the ANOVA indicated a significant effect, the means were compared by Bonferroni’s test. Differences were considered significant at P < 0.05. The results are presented as mean ± SD.

## References

[1] Martinez EA (2016). Recent advances toward the practical application of embryo transfer in pigs. Theriogenology.

[2] Martinez EA (2015). Nonsurgical deep uterine transfer of vitrified, *in vivo*-derived, porcine embryos is as effective as the default surgical approach. Sci. Rep..

[3] Cuello C (2010). Superfine open pulled straws vitrification of porcine blastocysts does not require pretreatment with cytochalasin B and/or centrifugation. Reprod. Fertil. Dev..

[4] Martinez EA (2014). Successful non-surgical deep uterine transfer of porcine morulae after 24 hour culture in a chemically defined medium. PLoS One.

[5] Stringfellow, D. A. Recommendations for the sanitary handling of *in vivo* derived embryos. In Manual of the International Embryo Transfer Society (eds Stringfellow, D. A. & Siedel, S. M.) 167–170. (International Embryo Transfer Society. IETS, Savoy, Illinois, USA, 1998).

[6] Martinez CA (2018). Simple storage (CO_2_-free) of porcine morulae for up to three days maintains the *in vitro* viability and developmental competence. Theriogenology.

[7] Trounson AO, Willadsen SM, Rowson LE, Newcomb R (1976). The storage of cow eggs at room temperature and at low temperatures. J. Reprod. Fertil..

[8] Lindner GM, Anderson GB, BonDurant RH, Cupps PT (1983). Survival of bovine embryos stored at 48 °C. Theriogenology.

[9] Ideta A (2013). A simple medium enables bovine embryos to be held for seven days at 4 °C. *Sci*. Rep..

[10] Harper MJ, Rowson LE (1963). Attempted storage of sheep ova at 7 °C. J. Reprod. Fertil..

[11] Anderson GB, Foote RH (1975). Development of rabbit embryos after storage at 10 °C. J. Anim. Sci..

[12] Nishijima K (2013). Delaying embryo development by storing at 4 °C for synchronization to recipients in microinjection technique in rabbits. Lab. Anim..

[13] Sakurai T, Kimura M, Sato M (2005). Temporary developmental arrest after storage of fertilized mouse oocytes at 4 °C: effects on embryonic development, maternal mRNA processing and cell cycle. Mol. Hum. Reprod..

[14] Takeo T (2010). Short-term storage and transport at cold temperatures of 2-cell mouse embryos produced by cryopreserved sperm. J. Am. Assoc. Lab. Anim. Sci..

[15] de Dios Hourcade J, Pérez-Crespo M, Serrano A, Gutiérrez-Adán A, Pintado B (2012). *In vitro* and *in vivo* development of mice morulae after storage in non-frozen conditions. Reprod. Biol. Endocrinol..

[16] Grau N (2013). Short-term storage of tripronucleated human embryos. J. Assist. Reprod. Genet..

[17] Niimura S, Ishida K (1980). Histochemical observation of lipid droplets in mammalian eggs during the early development. Jpn. J. Anim. Reprod..

[18] Nagashima H (1994). Removal of cytoplasmic lipid enhances the tolerance of porcine embryos to chilling. Biol. Reprod..

[19] Polge, C. The freezing of mammalian embryos: perspectives and possibilities. In *The Freezing of Mammalian Embryos* (eds Elliott, K. & Whelan, J.) 3–18. (Amsterdam: Elsevier, Excerpta Medica, 1977). 10.1002/9780470720332.ch2 244400

[20] Pomar FJ (2004). Development, DNA fragmentation and cell death in porcine embryos after 24 h storage under different conditions. Theriogenology.

[21] Lane M, Maybach JM, Hooper K, Hasler JP, Gardner DK (2003). Cryo-survival and development of bovine blastocysts are enhanced by culture with recombinant albumin and hyaluronan. Mol. Reprod. Dev..

[22] Uysal O, Korkmaz T, Tosun H (2005). Effect of bovine serum albumin on freezing of canine semen. Indian Vet. J..

[23] Van Thuan N, Wakayama S, Kishigami S, Wakayama T (2005). New preservation method for mouse spermatozoa without freezing. Biol. Reprod..

[24] Nang CF (2012). Bovine serum albumin: survival and osmolarity effect in bovine spermatozoa stored above freezing point. Andrologia.

[25] Whittingham DG (1974). The viability of frozen-thawed mouse blastocysts. J. Reprod. Fert..

[26] Rizos D (2003). Bovine embryo culture in the presence or absence of serum: implication for blastocyst development, cryotolerance, and messenger RNA expression. Biol. Reprod..

[27] Mucci N (2006). Effect of estrous cow serum during bovine embryo culture on blastocyst development and cryotolerance after slow freezing or vitrification. Theriogenology.

[28] Tominaga K, Iwaki F, Hochi S (2007). Conventional freezing of *in vitro*-produced and biopsied bovine blastocysts in the presence of a low concentration of glycerol and sucrose. J. Reprod. Dev..

[29] Men H, Agca Y, Critser ES, Critser JK (2005). Beneficial effect of serum supplementation during *in vitro* production of porcine embryos on their ability to survive cryopreservation by open pulled straw vitrification. Theriogenology.

[30] Lindner GM, Ellis DE (1985). Refrigeration of bovine embryos. Theriogenology.

[31] Angel MA (2014). The effects of superovulation of donor sows on ovarian response and embryo development after nonsurgical deep-uterine embryo transfer. Theriogenology.

[32] Angel MA (2014). An earlier uterine environment favors the *in vivo* development of fresh pig morulae and blastocysts transferred by a nonsurgical deep-uterine method. J. Reprod. Dev..

[33] Dobrinsky JR (2002). Advancements in cryopreservation of domestic animal embryos. Theriogenology.

[34] Cuello C (2004). Vitrification of porcine embryos at various developmental stages using different ultra-rapid cooling procedures. Theriogenology.

[35] Holm P, Booth PJ, Schmidt MH, Greve T, Callesen H (1999). High bovine blastocyst development in a static *in vitro* production system using SOFaa medium supplemented with sodium citrate and myo-inositol with or without serum-proteins. Theriogenology.

[36] Abe H, Yamashita S, Satoh T, Hoshi H (2002). Accumulation of cytoplasmic lipid droplets in bovine embryos and cryotolerance of embryos developed in different culture systems using serum free or serum-containing media. Mol. Reprod. Dev..

[37] Vajta G, Rienzi L, Cobo A, Yovich J (2010). Embryo culture: can we perform better than nature?. Reprod. Biomed. Online.

[38] Thompson JG (2000). *In vitro* culture and embryo metabolism of cattle and sheep embryos – a decade of achievement. Anim. Reprod. Sci..

[39] Francis GL (2010). Albumin and mammalian cell culture: implications for biotechnology applications. Cytotechnology.

[40] Baguisi A, Arav A, Crosby TF, Roche JF, Boland MP (1997). Hypothermic storage of sheep embryos with antifreeze proteins: Development *in vitro* and *in vivo*. Theriogenology.

[41] Clark KE, Squires EL, McKinnon AO, Seidel GE (1987). Viability of stored equine embryos. J. Anim. Sci..

[42] Cuello C (2007). The effectiveness of the stereomicroscopic evaluation of embryo quality in vitrified-warmed porcine blastocysts: an ultrastructural and cell death study. Theriogenology.

[43] Lindner GM, Wright RW (1978). Morphological and quantitative aspects of the development of swine embryos *in vitro*. J. Anim. Sci..

[44] Kovačič B, Taborin M, Vlaisavljević V (2018). Artificial blastocoel collapse of human blastocysts before vitrification and its effect on re-expansion after warming - a prospective observational study using time-lapse microscopy. Reprod. Biomed. Online.

[45] Marcos J (2015). Collapse of blastocysts is strongly related to lower implantation success: a time-lapse study. Human Reproduction.

[46] Hur YS (2011). Effect of artificial shrinkage on clinical outcome in fresh blastocyst transfer cycles. Clin. Exp. Reprod. Med..

[47] Iwayama H, Hochi S, Yamashita M (2011). *In vitro* and *in vivo* viability of human blastocysts collapsed by laser pulse or osmotic shock prior to vitrification. J. Assist. Reprod. Genet..

[48] Darwish E, Magdi Y (2016). Artificial shrinkage of blastocoel using a laser pulse prior to vitrification improves clinical outcome. J. Assist. Reprod. Genet..

[49] Wright, J. M. Photographic illustrations of embryo developmental stage and quality codes. In Manual of the International Embryo Transfer Society (eds Stringfellow, D. A. & Siedel, S. M.) 167–170. (International Embryo Transfer Society. IETS, Savoy, Illinois, USA, 1998).

[50] Petters RM, Wells KD (1993). Culture of pig embryos. J Reprod Fertil.

[51] Cuello C (2016). Effective vitrification and warming of porcine embryos using a pH-stable, chemically defined medium. Sci. Rep..

